# Global blood pressure screening during the COVID-19 pandemic: results from the May Measurement Month 2021 campaign

**DOI:** 10.1097/HJH.0000000000003488

**Published:** 2023-06-19

**Authors:** Thomas Beaney, Wei Wang, Markus P. Schlaich, Aletta E. Schutte, George S. Stergiou, Luis Alcocer, Jafar Alsaid, Alejandro Bimbo Diaz, Rafael Hernandez-Hernandez, Mohammad Ishaq, Jacek Jozwiak, Nadia Khan, Gaia Kiru, Harsha McCardle, Augustine Nonso Odili, Wook Bum Pyun, Cesar A. Romero, Jiguang Wang, Neil R. Poulter

**Affiliations:** a Imperial Clinical Trials Unit, and; b Department of Primary Care and Public Health, Imperial College London, London, UK; c Dobney Hypertension Centre, Medical School, Royal Perth Hospital Unit – University of Western Australia, Perth, Western Australia; d School of Population Health, University of New South Wales, The George Institute for Global Health, Sydney, Australia; e Hypertension in Africa Research Team/SAMRC Unit for Hypertension and CVD, North-West University, Potchefstroom, South Africa; f School of Medicine, Hypertension Center STRIDE-7 National and Kapodistrian University of Athens Third Department of Medicine, Sotiria Hospital, Athens, Greece; g Instituto Mexicano de Salud Cardiovascular, Tuxpan 16, Roma Sur, Cuauhtemoc, Mexico; h University of Queensland, Brisbane, Queensland, Australia; i Ochsner Medical Center, New Orleans, Louisiana, USA; j Department of Neuroscience and Behavioral Medicine, University of Santo Tomas Hospital, Manila, Philippines; k Hypertension and Cardiovascular Risk Factors Clinic, Dean of Health Sciences, Universidad Centro Occidental Lisandro Alvarado, Barquisimeto, Venezuela; l Karachi Institute of Heart Diseases, AGA Khan University Hospital, Karachi, Pakistan; m Department of Family Medicine and Public Health, Faculty of Medicine, University of Opole, Opole, Poland; n Department of Medicine, Center for Health Evaluation and Outcomes Sciences, University of British Colombia, Vancouver, Canada; o Khanda Ltd, London, United Kingdom; p Circulatory Health Research Laboratory, College of Health Sciences, University of Abuja, Abuja, Nigeria; q Division of Cardiology, Department of Internal Medicine, Seoul Hospital, College of Medicine, Ewha Womans University, Seoul, Korea; r Renal Division, Emory University School of Medicine, Atlanta, Georgia, USA; s Rujin Hospital, Shanghai Jiaotong University School of Medicine, Shanghai, China

**Keywords:** adults, awareness, blood pressure, COVID-19, hypertension, risk factor, screening, treatment

## Abstract

**Background:**

Raised blood pressure (BP) remains the biggest risk factor contributing to the global burden of disease and mortality, despite the COVID-19 pandemic. May Measurement Month (MMM), an annual global screening campaign aims to highlight the importance of BP measurement by evaluating global awareness, treatment and control rates among adults with hypertension. In 2021, we assessed the global burden of these rates during the COVID-19 pandemic.

**Methods:**

Screening sites were set up in 54 countries between May and November 2021 and screenees were recruited by convenience sampling. Three sitting BPs were measured, and a questionnaire completed including demographic, lifestyle and clinical data. Hypertension was defined as a systolic BP at least 140 mmHg and/or a diastolic BP at least 90 mmHg (using the mean of the second and third readings) or taking antihypertensive medication. Multiple imputation was used to impute the average BP when readings were missing.

**Results:**

Of the 642 057 screenees, 225 882 (35.2%) were classified as hypertensive, of whom 56.8% were aware, and 50.3% were on antihypertensive medication. Of those on treatment, 53.9% had controlled BP (<140/90 mmHg). Awareness, treatment and control rates were lower than those reported in MMM campaigns before the COVID-19 pandemic. Minimal changes were apparent among those testing positive for, or being vaccinated against COVID-19. Of those on antihypertensive medication, 94.7% reported no change in their treatment because of the COVID-19 pandemic.

**Conclusion:**

The high yield of untreated or inadequately treated hypertension in MMM 2021 confirms the need for systematic BP screening where it does not currently exist.

## INTRODUCTION

Best evidence suggests that since 2019 and despite the ravages of the COVID-19 pandemic, raised blood pressure (BP) continues to be the biggest contributor to global morbidity and mortality [1,2]. Furthermore, previously reported trends for improvement in hypertension management may have stalled during that time [3–6]. The global number of people living with hypertension in 2019 is estimated to be over 1.2 billion, which is double that in 1990 [2]. It is estimated that raised BP caused approximately 30 000 deaths per day in 2019 [2], which exceeds the global daily death rate because of COVID-19 at any stage of the pandemic [7].

Whilst new treatments and effective vaccines are likely to further reduce the adverse impact of COVID-19 on global disease burden in the future [8], the adverse impact of raised BP is likely to continue to increase [3]. This is in part because routine screening and management of chronic conditions such as raised BP have been subsumed by competing demands on resources and staff during the COVID-19 pandemic [5].

Meanwhile, the COVID-19 pandemic notwithstanding, as the world's population size grows, and with mean age and rates of obesity increasing, the burden of raised BP inevitably continues to worsen. In 2017, the global May Measurement Month (MMM) campaign was initiated [9] by the International Society of Hypertension (ISH) in recognition of the fact that low rates of BP control amongst the world's hypertensive population critically reflected low rates of awareness and diagnosis of the condition. Hence, the primary aim of MMM is to raise awareness of the importance of BP measurement at the individual and population level and, meanwhile, to act as a pragmatic interim solution to the shortfall in BP screening programmes around the world.

In three MMM campaigns carried out in 2017, 2018 and 2019, BP screening of over 4.2 million adults from more than 100 countries detected almost one million people with untreated or inadequately treated hypertension [9–11]. In 2020, the MMM campaign was deferred because of the COVID-19 pandemic. In 2021, by extending the screening period beyond May, we were able to continue the MMM series in parts of the world where it was safe to do so because of the reduced threat and distractions caused by COVID-19. We report the findings of MMM in 2021, a contemporary globally standardized BP screening survey carried out in the remarkable and unique circumstances of the COVID-19 pandemic, which provided the opportunity to evaluate the impact of aspects of the pandemic on BP measurement and management around the world. In addition, using data collected from each screenee in a short questionnaire, we evaluate to what extent BP parameters were impacted by the demographic variables, medical conditions and lifestyle activities reported.

## METHODS

### Study design

MMM21 is a cross-sectional survey of BP in adults (≥18 years old) recruited through convenience sampling. Screening sites were set up around the world, following a single protocol and study design (see https://maymeasure.org/). In 2021, the period of screening was extended from May to November to allow sites’ flexibility to adapt to local COVID-19 control measures.

One hundred and seven countries were contacted via international and national hypertension and cardiovascular disease societies or through networks established in previous MMM campaigns. Each country appointed a national lead investigator(s), responsible for the set-up and coordination of screening sites within each country and for obtaining ethics approval, if required.

Screening sites were established in a range of locations, including healthcare settings, public spaces, workplaces, and COVID-19 vaccination centres. The campaign was promoted centrally by MMM, the ISH, and the World Hypertension League through newsletters and social media. Campaigns were also promoted locally through television, radio, social media and celebrity endorsements. Volunteer staff were recruited locally by national investigators and trained in BP measurement through written materials and training videos accessible on the MMM website (see https://maymeasure.org/) or through locally implemented in-person training. Where needed, BP machines were made available to sites courtesy of a donation of 3844 validated upper-arm-cuff BP devices (M2 and M3 Basic models) to MMM by OMRON Healthcare.

Adult participants were recruited opportunistically at screening sites and were included after giving informed consent to participate. Three BP and pulse rate measurements were taken from participants at 1 min intervals, after being seated for at least 5 min. An anonymized questionnaire was also completed, including data on demographics, medical history, lifestyle and weight (see Supplementary Appendix, Page 3). Weight was reported by the participants or measured if equipment was available.

Data were entered onto an MMM app available for mobile and desktop devices in nine different languages, and with offline capabilities to store data for screening sites with limited connectivity. Where the app was not used, data were collected via spreadsheets. A small number of submissions were also received directly by participants (1567) through an ‘MMM at home’ submission site on the MMM website.

Hypertension was defined as SBP at least 140 mmHg and/or DBP at least 90 mmHg, based on the average of the second and third BP readings, or the taking of antihypertensive medication. Controlled BP was defined as SBP less than 140 mmHg and DBP less than 90 mmHg in participants taking antihypertensive medication. In participants measuring their BP at home, a lower threshold of 135/85 mmHg was used for defining hypertension and control in keeping with current guidelines [12,13]. Participants with untreated or uncontrolled hypertension were provided with advice on follow-up, which was tailored to the local healthcare setting, along with a summary of advice on lifestyle and dietary ‘Ten Top Tips’ to lower BP (see Supplementary Appendix, page 4)

### Statistical analysis

Data were submitted from screening sites via the MMM app or spreadsheets and were collated and cleaned centrally. Prespecified data cleaning rules, including cut-off values for continuous data, were applied (see Supplementary Appendix, page 5). Participants without at least one valid SBP and DBP reading were excluded from the study.

National economic income was defined according to the World Bank classification 2021 except for Venezuela for which the latest available 2019 classification was applied [14]. Geographic regions were classified according to those used in previous MMM campaigns, based on minor modifications to the United Nations classification [15]. Age and sex standardization was applied based on single-year age estimates of the WHO world-standard population, and assuming a 1 : 1 male-to-female ratio [16].

For participants missing one or two BP readings, multiple imputation using chained equations was used to estimate the mean of the second and third readings, under an assumption that BP readings were missing-at-random. We followed the approach used in previous MMM analyses, for which results were shown to be robust to alternate model specifications [9–11]. Two separate imputation models were constructed. The first ‘full’ model included only individuals with no missing data on age, sex, ethnicity and use of antihypertensive medication. Included in the model were age and sex (along with an interaction term), hypertension awareness, weight, weight-squared, pregnancy, history of hypertension in pregnancy, diabetes, myocardial infarction, stroke, alcohol, smoking, screening site, the three pulse rate readings, along with country income and region. For those participants missing at least one of age, sex, ethnicity and antihypertensive medication, a ‘partial’ model was run, imputing based only on the available SBP and DBP readings. The mean of the second and third BP and pulse rate readings were included in both models along with the individual readings. Fifteen imputations were created, corresponding approximately to the percentage of missing BP data and was deemed sufficient based on Monte Carlo errors for the estimates less than 10% of the standard errors [17,18]. Further details of the imputations are found in the Supplementary Appendix (page 7).

Associations were evaluated between parameters of BP management as dependent variables and demographics (e.g. by age, sex and BMI) self-reported medical conditions (e.g. diabetes, hypertension in a previous pregnancy) and lifestyle activities (e.g. exercise) as reported in the study questionnaire (see Supplementary Appendix, page 3). In 2021, the MMM questionnaire allowed an evaluation of the impacts of having a positive COVID-19 test and having received a COVID-19 vaccination on BP levels and whether the pandemic had impacted on the treatment of hypertension. In addition, the questionnaire included for the first-time questions on the use of hormonal contraception and hormone replacement therapy (HRT), adherence to BP-lowering medications, costs of treatment, exercise and years of education. Associations between these variables and BP parameters (as dependant variables) were also evaluated, because whilst each of these variables have been reported to impact on BP levels, rarely, if ever, have such associations been shown, in such large samples arising from all over the world.

Measures of all these associations were examined using two-level hierarchical linear models, including country of screening as a random intercept to account for clustering, and assuming fixed slopes. Separate models were run for SBP and DBP. Only participants with non-missing data in age, sex, ethnicity and antihypertensive medication were included in the analyses (i.e. excluding those imputed via the ‘partial’ model). We included age and sex (along with an interaction between age and sex) and antihypertensive medication as confounders in the models, given the known strong relationships between these factors and BP [9]. Models including known hypertension and antihypertensive medication use as exposures were carried out for the subset of participants defined as hypertensive and adjusted for age and sex alone. Age was modelled as a restricted cubic spline with five knots, to allow for flexibility in modelling the relationship with BP. Data were analysed using Python version 3.7.4, Pandas version 1.3.4 and Stata version 16.1 (StatCorp, College Station Texas, USA).

Anonymized participant data from MMM are available for research purposes with approval from MMM with a Data Use Agreement in place (for more information, see https://maymeasure.org or e-mail the corresponding author). Analytic codes are available on request to the corresponding authors.

## RESULTS

### Screening sites

Data were received on 651 008 participants from 54 countries (see Supplementary Appendix Table S2). After data cleaning, data from 642 057 participants were included in the analysis, 80 134 (12.5%) of which were submitted through the MMM app. The full range of national income classes were included, with 6.6, 26.2, 65.4 and 1.9% from low-income, lower middle-income, upper middle-income and high-income countries, respectively. There was a wide geographical distribution of participants, but the majority were screened in East Asia (34.7%) and the Americas (24.4%) with relatively small numbers from Europe and North America (Table 1).

**TABLE 1:** Worldwide and regional distribution of participants by age, sex and use of antihypertensive medication

		Female		Male	
Region	Total participants	Total	Mean age (years)	Total	Mean age (years)
East Asia	222 614 (34.7%)	114 832 (51.6%)	46.5	107 722 (48.4%)	47.6
Americas	156 513 (24.4%)	93 310 (59.9%)	49.6	62 528 (40.1%)	51.6
South-east Asia and Australasia	89 631 (14.0%)	44 339 (49.9%)	44.6	44 288 (49.9%)	41.8
Sub-Saharan Africa	73 880 (11.5%)	35 422 (48.0%)	41.4	38 300 (51.9%)	39.8
South Asia	59 946 (9.3%)	22 637 (37.9%)	40.8	37 152 (62.1%)	43.7
Europe	38 327 (6.0%)	22 719 (59.3%)	50.1	15 550 (40.6%)	52.0
Northern Africa and Middle East	1146 (0.2%)	706 (61.6%)	46.5	440 (38.4%)	48.0
**Worldwide**	**642** **057**	**333** **965 (52.2%)**	**46.4**	**305** **980 (47.8%)**	**46.4**

Most screening (65.7%) took place in healthcare settings (hospitals, clinics or pharmacies), with 15.4% at outdoor public areas, 6.1% at workplaces, 5.0% at indoor public areas, 2.9% at COVID-19 vaccination sites and 0.2% through the ‘MMM at Home’ website.

### Participant characteristics

Response rates varied to different survey questions, with over 99% of participants having age and sex recorded, but lower response rates for other questions, such as date of last BP measurement (53.6% completed) and new survey questions such as years of education (66.2%). The mean (SD) age of participants overall was 46.4 (16.5) years, but mean age varied by region with the youngest in sub-Saharan Africa (40.5 years) and the oldest in Europe (50.8 years) (Table 1). Overall, more participants were female (52.2%) than male (47.8%), but significant variation in sex distribution was apparent across regions, with the lowest proportion of females (37.9%) in South Asia and the highest (61.6%) in Northern Africa and the Middle East.

Participant characteristics are presented in the Supplementary Appendix (Table S3). Of the 78.8% participants with ethnicity recorded, the majority were East or South-east Asian (52.6%), Black (14.9%) or South Asian (13.6%). Of participants with recorded data for each variable, 24 382 (5.2%) reported diabetes (type 1 or type 2), 10 535 (2.2%) reported a history of myocardial infarction, 6230 (1.3%) reported a history of stroke and 48 385 (9.9%) were current smokers. The majority (87.4%) drank alcohol never or rarely, with 9.9% at least monthly and 3.9% at least weekly. Of women, 1.5% were pregnant, 2.4% were using hormonal contraception, 0.6% were taking HRT and 2.1% reported a history of hypertension in a previous pregnancy. 95.4% of participants had completed at least 1 year of education, with 33% completing over 12 years. 27.7% reported meeting the WHO target of at least 150 min of moderate exercise or 75 min of vigorous exercise per week. A small percentage (5.2%) reported a previous positive COVID-19 test, and 45.2% reported at least one previous COVID-19 vaccination.

### Blood pressure differences

In total, 548 983 (85.5%) participants had three BP readings recorded, with 8.8% having two and 5.7% only one reading. Among participants with all three readings, mean SBP and DBP and hypertension rates fell across subsequent measurements from 126.3/80.4 mmHg with 32.9% hypertensive (first reading) to 123.5/78.6 mmHg with 29.1% hypertensive (third reading). The mean of the second and third BP reading resulted in the lowest percentage of those screened meeting the criteria for hypertension (29.1%) (See Supplementary Appendix, Table S4). Mean DBP was consistently higher in men than women, and mean SBP was higher in men until the age of about 75 years when women tended to have higher mean SBPs (See Supplementary Appendix, Figure S1).

### Hypertension, awareness, treatment and control

Multiple imputation was used to impute the average of the second and third BP readings for 89 977 participants with one or two missing BP readings (22 592 from the full model and 67 385 from the partial model).

Worldwide, of 642 057 participants, 225 882 (35.2%) were classified as having hypertension. Of those with hypertension, 128 238 (56.8%) were aware of having hypertension and 113 580 (50.3%) were on antihypertensive medication. Of those on antihypertensive medication, 61 231 (53.9%) had controlled BP, and of all hypertensive participants, 27.1% had controlled BP. Of all participants not taking antihypertensive medication, 112 302 (21.3%) had hypertension. In total, 164 651 (25.6%) of those screened had either untreated, or inadequately treated hypertension. There were large regional differences in rates of hypertension awareness, treatment and control (Table 2; corresponding confidence intervals are given in Supplementary Appendix Table S6).

**TABLE 2:** Worldwide and regional numbers with hypertension and percentages aware, on treatment and controlled (of all 642 057 participants)

Region	Number with hypertension	Percentage with hypertension	Percentage of hypertensive participants aware	Percentage of hypertensive participants on medication	Percentage of those on medication with controlled BP	Percentage of all hypertensive participants controlled
East Asia	59 133	26.6%	32.4%	30.6%	61.5%	18.8%
Americas	60 475	38.6%	72.9%	59.5%	55.0%	32.7%
South-east Asia and Australasia	36 866	41.1%	49.6%	45.1%	46.8%	21.1%
Sub-Saharan Africa	27 951	37.8%	66.7%	63.6%	51.9%	33.0%
South Asia	21 366	35.6%	64.6%	55.6%	57.7%	32.1%
Europe	19 518	50.9%	71.2%	66.0%	49.1%	32.4%
Northern Africa and Middle East	573	50.0%	69.9%	65.4%	39.7%	26.0%
**Worldwide**	**225** **882**	**35.2%**	**56.8%**	**50.3%**	**53.9%**	**27.1%**

Confidence intervals for the percentages are provided in the Supplementary Appendix Table A.

Sensitivity analyses showed similar average SBPs and DBPs and proportions with raised BP in the complete case analysis compared with each of the imputation models (see Supplementary Appendix, Table S5).

Overall, age had a major impact on rates of hypertension, which rose from 13.8% in those aged 18–29 years to 62% in those aged 70+ years (see Supplementary Appendix, Table S7). There were also significant differences according to sex, with a higher percentage of hypertension in men in age groups less than 70 years, but a higher percentage in women aged 70 years of age (see Supplementary Appendix, Table S8). Awareness, treatment and control rates were all higher in women compared with men (see Supplementary Appendix, Table S9).

After standardization to the WHO world age-standard population, of the 636 658 (99.2%) with age and sex recorded, 31.1% globally were classified as hypertensive (see Supplementary Appendix, Table S10). Differences in the percentages with hypertension between regions were attenuated following standardization, reflecting the uneven distribution of age and sex across regions. Nevertheless, a range from 23.5% in East Asia to 45.4% in Northern Africa and the Middle East remained. There was a trend towards lower DBPs in higher income countries (*P* = 0.003) but no significant trend in SBP (*P* = 0.125).

### Impact of COVID-19

Of the 74.1% of participants with recorded data on testing for COVID-19, 24 614 (5.2%) reported a history of a positive COVID-19 test, and of the 72.7% with recorded data on COVID-19 vaccination, 45.2% had received at least one COVID-19 vaccination. Of those on antihypertensive medication, the majority (94.7%) reported no change in their BP treatment as a result of the COVID-19 pandemic, but 1010 (1.5%) reported that their usual drugs were unavailable and 646 (0.9%) reported they were unable to access their healthcare provider.

**FIGURE 1:**
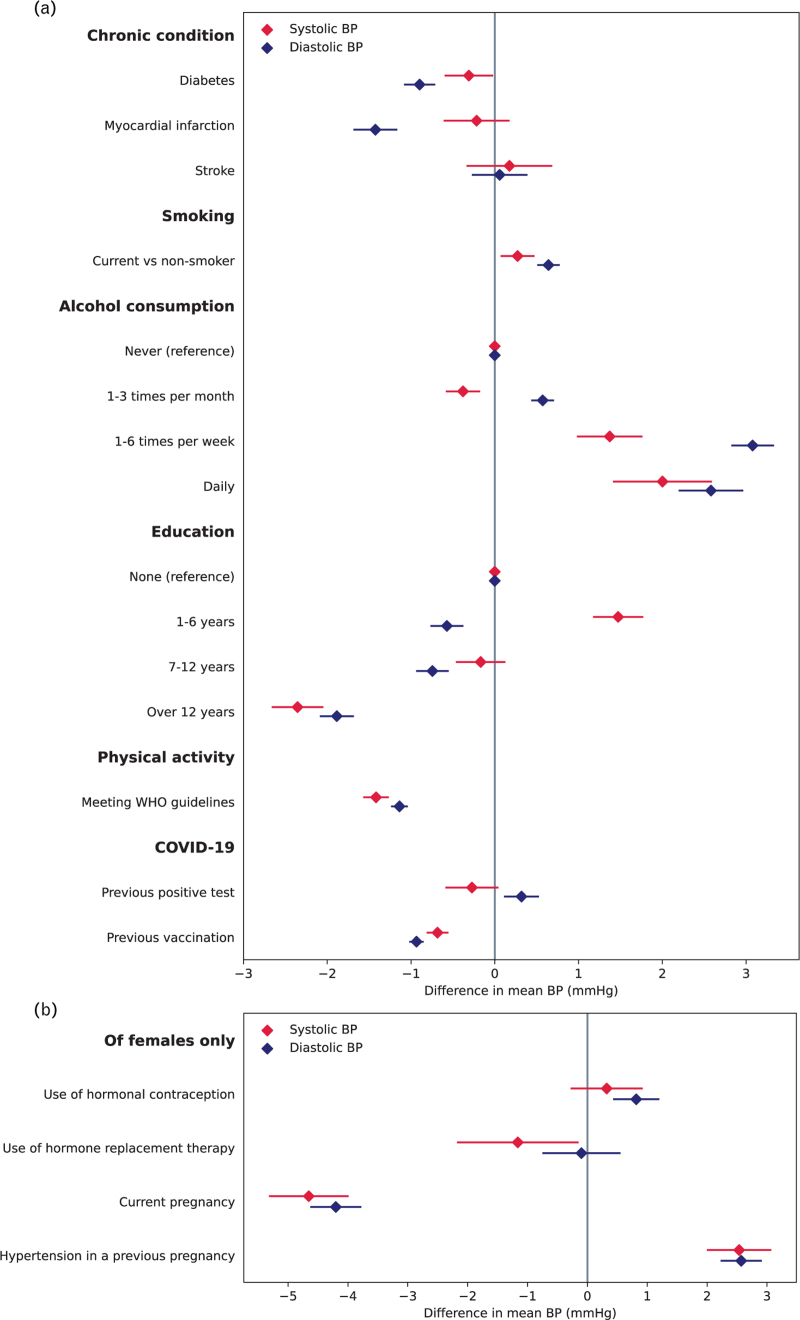
Difference in average SBP and DBP in participants with different risk factors, for whole population (panel a) and of female participants only (panel b). Results from linear mixed models adjusted for age, sex and antihypertensive medication (panel a), or age and antihypertensive medication (panel b).

After adjustment for age, sex and antihypertensive medication use, there was no significant difference in average SBP in those with a previous COVID-19-positive test compared with those without, but average DBP was 0.32 [95% confidence interval (CI) 0.10 – 0.53] mmHg higher. In people with a previous COVID-19 vaccination, SBP and DBP were both significantly lower than in those without vaccination (Fig. 1 and Appendix Table S12). These associations remained after additional adjustment for participant years of education.

A total of 14 779 (24.2%) participants from the Philippines had their BP screened at a COVID-19 vaccination centre. After age and sex standardization, those screened at vaccination centres had significantly higher SBPs (136.7 vs. 127.2 mmHg) and higher pulse rates (87.8 vs. 81.1 bpm) but similar DBPs (81.5 vs. 81.8 mmHg) compared with participants who had their BPs screened at other sites in the Philippines.

### May Measurement Month at Home

Of 1567 participants recruited via the MMM at Home website, 1474 (94.1%) were from the Philippines. Among 61 129 participants in the Philippines, average age and sex-standardised BPs were lower among participants screened at home (121.9/78.8 mmHg) compared with those not screened at home (129.3/81.6 mmHg). Despite lower average BPs, the home BP group were more likely than other participants to be hypertensive, albeit using a lower diagnostic threshold for home readings (52.9 vs, 42.4%) and hypertensive participants had significantly higher standardized rates of awareness (92.4 vs. 56.1%), treatment (90.7 vs. 42.9%), and control (57.1 vs. 24.4%).

### Medication use and hypertension control

**FIGURE 2:**
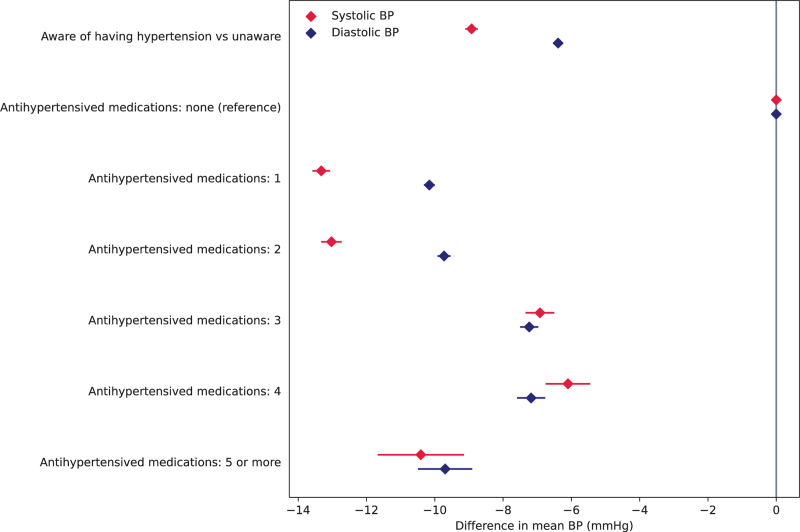
Difference in average SBP and DBP in participants with hypertension for those aware compared with those unaware and those taking compared with those not taking antihypertensive medication. Results from linear mixed models adjusted for age and sex.

Among screenees reporting on their antihypertensive medication, 58.1% reported taking a single class of agents and 27.5, 10.3, 3.3 and 0.8% reported taking two, three, four or five or more, respectively. Hypertensive participants on medication had significantly lower average SBPs (12.5 mmHg lower, *P* < 0.001) and DBPs (9.6 mmHg lower, *P* < 0.001) compared with hypertensive participants not on medication. Compared with hypertensive participants not on medication, the reported number of BP-lowering agents used was associated with lower average SBP and DBP but with a trend towards smaller BP differences with increasing numbers of agents (Fig. 2 and Supplementary Appendix, Table S11).

Of the 67% of those on medication who reported regularity of use, 73% were taking their medication regularly. Of those who did not take their medication regularly, 71.9% reported taking them only when needed, 14.6% said they were too expensive, 6% reported they forgot, 4.5% reported that availability was not easy, whereas 1.6% preferred alternative medications and only 1.5% reported not taking their medication because of side effects. Of those taking medication regularly, 50.8% had controlled BP (average BP 136.8/84.3 mmHg), compared with 53.3% (average BP 134.1/82.9 mmHg) in those that were not taking medication regularly (*P* value for difference <0.001).

Of those on antihypertensive medication, 25.8% reported paying nothing for consultations or medications, 37.6% paid part of the costs and 36.6% paid all costs. Of those paying nothing, 55.8% were controlled, compared with 55.4% in those paying part, and 49.8% in those paying all costs (*P* < 0.001).

### Lifestyle and other factors

After adjustment for age, sex, and antihypertensive medication, people meeting the WHO-recommended exercise target had statistically significantly lower SBPs and DBPs (1.4/1.1 mmHg lower, *P* < 0.001) compared with those who did not. Smokers had higher BPs compared with nonsmokers (*P* < 0.01), with a trend towards lower BPs in those with more years of education (overall *P*_trend_ <0.001) and higher BPs with increasing frequency of alcohol intake (overall *P*_trend_ <0.001) (Fig. 1 and Supplementary Appendix, Table S13). Participants with diabetes had significantly lower average SBP and DBPs than those without, whilst participants with a previous myocardial infarction had significantly lower diastolic but not systolic average BPs and participants with a previous stroke had no significant difference in average BPs (Fig. 1 and Supplementary Appendix, Table S14).

After adjustment for age and antihypertensive medication use, women who were pregnant at the time of screening had significantly lower SBPs and DBPs than those not pregnant, whereas those with a history of hypertension in pregnancy had significantly higher BPs than those with no such history. Women using HRT had significantly lower SBP (1.2 mmHg lower, *P* = 0.025) but no significant difference in DBP than non-users, whereas those using hormonal contraception had higher DBP (0.8 mmHg higher, *P* < 0.001) but no significant difference in SBP compared with nonusers (Fig. 1 and Supplementary Appendix, Table S15) after adjustment for age and medication use.

Amongst participants not taking antihypertensive medication, a strong linear association was shown between increasing heart rate and DBP, with an inverse J-shaped relationship apparent between heart rate and SBP (Supplementary Appendix, Figure S2 and Table S16).

## DISCUSSION

The COVID-19 pandemic seriously affected the annual MMM campaign causing cancellation in 2020, and in 2021 the potential screening time was extended from May to November 2021, to maximize screening opportunities. Moreover, only 54 countries participated compared with almost 100 countries in previous years [9–11]. The result was that some regions of the world, particularly North America, Europe and South Asia were underrepresented. Nevertheless, almost two-thirds of a million adults predominantly of East or South-East Asian ethnicity took part in 2021 of whom about one-third (35.2%) were classified as hypertensive.

Despite some underrepresentation of data from some parts of the world, the large number of screenees involved raises the potential problem of interpreting small but statistically significant differences in variables (usually BP), which may not be clinically significant at the individual level. As previously described, however, these small BP differences may be important and impactful at a population level [19].

Among just over one quarter of a million hypertensive screenees, only about half (56.8%) were aware of their condition, and one half (50.3%) were on treatment. Of those on treatment for hypertension, only 53.9% were controlled to the conservative target of less than 140/90 mmHg. This is reminiscent of the ‘rule of halves’ reported over 50 years ago in the USA [20]. Taken at face value, this would suggest that many countries in the world have only achieved rates of awareness, treatment and control that the United States was achieving 50 years ago. Nevertheless, in the last half century, rates of awareness, treatment and control have improved in some parts of the world [21,22], but even in high-income countries, truly high rates have not been achieved [23]. The control rate amongst those treated in MMM 2021 fell to 25%, if the more contemporary guideline-recommended target [12,13] of less than 130/80 mmHg was applied. Furthermore, the majority (58.1%) of those on antihypertensive medication were taking only a single agent indicating significant scope for more easily attained BP control with single-pill combination therapies as advised by the latest hypertension guidelines [12,13].

Despite the extended time of year and change in circumstances under which MMM 2021 took place in a reduced number of countries, the age and sex distribution of participants and the headline results of the global cascade of hypertension care are consistent with the combined results of the previous three campaigns. Although awareness, treatment and control rates were lower than in the three previous campaigns [9–11] (Table 3), which may reflect the impact of the COVID-19 pandemic, direct comparisons across the 4 years maybe misleading given the non-random sampling used each year.

**TABLE 3:** Comparison of key statistics from MMM21 compared with previous MMM campaigns

MMM campaign	Total participants	Mean (SD) age (years)	Percentage female : male	Percentage with hypertension	Percentage of hypertensive participants aware	Percentage of hypertensive participants on medication	Percentage of those on medication with controlled BP	Percentage of all hypertensive participants controlled
MMM17	1 201 570	44.9 (16.9)	54.0% : 45.0%	34.9%	N/A	57.8%	53.7%	31.0%
MMM18	1 504 963	45.3 (17.0)	52.4% : 46.7%	33.4%	59.5%	55.3%	60.0%	33.2%
MMM19	1 508 130	45.8 (17.0)	51.6% : 48.4%	34.0%	58.7%	54.7%	57.8%	31.7%
MMM21	642 057	46.4 (16.5)	52.2% : 47.8%	35.2%	56.8%	50.3%	53.9%	27.1%

MMM, May Measurement Month.

a Percentages for ‘Other’ and missing data not shown.

The impact of the COVID-19 pandemic on BP control remains uncertain, with scarce evidence particularly in lower income settings. An interrupted time series of over 100 000 people with hypertension in the United States found worsening of BP control in the first 8 months of the pandemic [24], and a UK-based study found fewer people initiated on antihypertensive medication than expected in the first year of the pandemic [25]. In contrast, a study of 50 000 adults with hypertension in Brazil found no evidence of a worsening of BP control in either treated or untreated individuals [26]. Although in our study, we are unable to determine changes over time, reassuringly, only about 1 in 20 of those taking BP-lowering medications reported any change in their treatment because of COVID-19. From among almost half a million participants who recorded data on testing for COVID-19, there was no significant difference in SBP and only a minimally higher DBP (albeit statistically significant) between those who reported testing positive and those without a positive test. However, both SBP and DBP were significantly lower among those vaccinated compared with the unvaccinated respondents, an association, which remained after further adjustment for years of education. To our knowledge, there is no definitive evidence of any long-term adverse impact on BP levels in association with either being infected or vaccinated for COVID-19 [3,27]. The small reductions in BP observed among those vaccinated may reflect confounding but are compatible with other data [27] in showing no significant long-term increase in BP levels because of vaccination.

The small subsample of 1474 adults screened at home in the Philippines had as expected [28] lower BPs than the rest of the Philippines population screened but had a higher rate of hypertension (using the lower diagnostic threshold recommended for home recordings [12,13]). This presumably reflects a population who are more likely to be hypertensive, and to be on treatment and thereby have higher control rates. By contrast, the large number of participants screened in vaccination centres in the Philippines had significantly higher SBPs and pulse rates than the overall Philippines population, which may reflect the stress associated with vaccination procedure.

Once again, the average of the second and third BP readings gave the most conservative estimate of hypertension, with implications for optimal practice when single-session BP screening is carried out.

Associations with average BP levels and various coexisting conditions and risk factors were similar to those observed in previous years, being higher in smokers, those with higher body weight, regular alcohol intake and a history of hypertension in pregnancy but lower among pregnant women and those with a previous history of MI.

Of the variables investigated for the first time in MMM 2021, there was a significant trend of lower BPs with increasing years of education and more aerobic exercise in keeping with well established associations between lower socioeconomic status and lack of exercise with higher BPs [29–31]. Women using HRT or oral contraception showed minimal differences in BP compared with non-users, albeit differences were significant for systolic and diastolic respectively, in keeping with the established small effects of these medications [32,33].

Amongst respondents reporting reasons for their nonadherence with BP-lowering therapy, side effects were rarely involved (1.5%), whilst equally surprisingly, 72% reported only taking their treatment when needed. Whether this reflects poor communication between prescriber and recipient or that the participants were conserving the drugs for financial reasons is not discernible, whilst 15% reported that drug cost caused their non-adherence. Paradoxically, those reporting good adherence to therapy had higher average BPs than those reporting non-adherence, possibly reflecting the greater need for treatment of the former group.

In keeping with previous MMM data [11], among those not taking BP-lowering medication, the direct relationship between increasing pulse rate and DBP contrasts with that between increasing pulse rate and SBP where only pulse rates above 90 bpm were associated with any increment in SBP. Other than the MMM databases, to our knowledge, this apparent disparity in the relationship between SBPs and DBPs with pulse rates has not been reported previously [34].

### Limitations

The true prevalence of hypertension in each country could not be evaluated given that, by design, nationally representative samples were not targeted, and in several parts of the world, such as Europe and North America, the sample sizes were relatively small. However, the primary aim of the study to raise awareness at the individual level was achieved by identifying almost 165 000 adults with untreated or inadequately treated hypertension and at the population level, via the extensive media and social media campaigns, which promoted MMM activities around the world.

Without a longitudinal component to the study, the definitive diagnosis, follow-up and outcomes of those classified as hypertensive participants in the MMM database cannot be confirmed but plans to address this shortcoming are in progress for future MMM surveys. Not all questions included in the survey were reported by all respondents, particularly for those newly added in MMM21, but 85.5% of participants did provide all three BP readings and overall response rates were sufficiently large to provide robust numbers to address the questions under investigation. Furthermore, given high rates of reporting of age and sex, missing responses to other survey questions are likely explained by questions not being asked systematically at the screening site, rather than participants declining to answer questions and so are likely to be missing at random with respect to participants. The lack of representative samples in each country may give rise to the misconception that the associations recorded with various BP parameters are not valid. However, that is not the case, especially given the large total sample arising from a very wide range of countries, regions, ethnicities and socioeconomic strata [35].

A potential limitation of the MMM campaign in 2021 was the reduced sample size compared with previous years – a direct result of the COVID-19 pandemic. The total number screened coming from 54 countries was approximately half that screened in MMM18 and MMM19, which came from 89 and 92 countries, respectively. However, even with the reduced participation, of two-thirds of a million adults in 2021, with the exception of previous MMM campaigns, MMM21 remains the largest contemporary standardized annual screening programme of BP levels around the world. Furthermore, it allowed MMM to collate unique information regarding COVID-19 vaccination and investigate the possibilities of collecting home BP readings in some countries.

### Strengths

Despite being smaller than the previous three campaigns, MMM21 identified 165 000 adults who had either untreated or inadequately treated raised BP. Those identified were given advice on how to lower their BPs and on how best to seek further follow-up of their condition. In parallel to these potential benefits for individuals, the multimedia promotional campaigns in over 50 countries worldwide enhanced awareness of the importance of BP measurement at the population level. We are hopeful that in MMM22, the higher numbers of countries and participants involved in previous campaigns will be restored.

### Perspectives

Since 2017, MMM campaigns, despite inactive in 2020 and only partially active in 2021, have screened the BPs of almost five million adults. Despite the large number of deaths worldwide resulting from the COVID-19 pandemic, raised BP causes the deaths of approximately 30 000 people per day [1], significantly higher than that from COVID-19. Meanwhile, we were unable to demonstrate any clear impact on BP levels of having tested positive or being vaccinated for COVID-19 nor to our surprise was hypertension management reportedly affected for the vast majority of those on treatment for hypertension whilst at the same time, global hypertension control rates were significantly lower in 2021 compared with previous years (Table 3). It is, therefore, more, rather than less vital to emphasize the importance of improving the detection of the biggest single contributor to global disease burden and mortality. Nothing, with the exception of prevention strategies (which are less easily achieved in a short time period) can contribute to improving the hypertension care cascade compared with enhancing the detection of raised BP, which, if translated into effective treatment, could prevent millions of major adverse cardiovascular events. By virtue of its opportunistic study design and volunteer workforce, MMM provides a simple, pragmatic and inexpensive way to contribute to reducing the shortfall in BP screening programmes in many countries and allows a rapid evaluation of variables impacting BP levels around the world.

## ACKNOWLEDGEMENTS

Our sincere thanks to Omron for the donations of blood pressure (BP) devices, World Hypertension League (WHL), and Professor Daniel T. Lackland for endorsing the extension of World Hypertension Day to May Measurement Month (MMM) and to Ranjit Rayat (Editing Assistant) for her superb, dedicated effort and to all volunteer staff and participants.

Sources of funding: May Measurement Month (MMM) was an initiative of the International Society of Hypertension. MMM 2021 was generously supported by Servier through the Institut la Conference Hippocrate and USASCP. As a supporter of the study, Servier had no role in study design, data collection, data analysis, data interpretation or writing of the report. The first and corresponding authors had full access to all the data in the study and had final responsibility for the decision to submit for publication.

### Conflicts of interest

T.B., A.E.S., G.S.S., J.A., A.B.D., R.H.H., M.I., N.K., G.K., H.M., A.N.O., W.B.P. and C.A.R. have nothing to declare. M.P.S. has received grants/travel support/honoraria from Abbott, Medtronic, Boehringer Ingelheim, Novartis and ReCor. W.W. was supported, in part, by a scholarship from the China Scholarship Council (202006260295). L.A. has received lecture and advisory fees from Servier and Omron. J.J. has received consulting fees and research support from Servier, Boehringer-Ingelheim, Teva, Amgen and Zentiva. J.W. reports having received grants from Novartis and Omron, and lecture and consulting fees from Novartis, Servier and Viatris. N.R.P. has received financial support from several pharmaceutical companies which manufacture blood pressure (BP)–lowering agents, for consultancy fees (Servier), research projects and staff (Servier, Pfizer) and for arranging and speaking at educational meetings (AstraZeneca, Lri Therapharma, Napi, Servier, Sanofi, Eva Pharma and Pfizer). He holds no stocks and shares in any such companies.

## Supplementary Material

Supplemental Digital Content
