# Supplementary material for: Global blood pressure screening during the COVID-19 pandemic: results from the May Measurement Month 2021 campaign
Source: J Hypertens. 2023 Jun 19;41(9):1446–55. doi: 10.1097/HJH.0000000000003488 (PMC10399936; doi:10.1097/HJH.0000000000003488)
Supplement: Supplemental Digital Content [file jhype-41-1446-s001.pdf]

Supplemental Material

Global blood pressure screening during the COVID-19 pandemic: results from the May Measurement Month 2021 campaign

Contents

MMM 2021 questionnaire p.3

MMM 2021 'Top 10 Tips' p.4

Data Cleaning Rules p.5

Statistical methods p.7

References p.8

Supplementary results p.9

…Tables p.9

…Figures p.24

MMM21 DATA CAPTURE FORM

PLEASE COMPLETE IN BLOCK CAPITALS ONLY, IN BLACK INK AND INSERT ONLY X IN THE CHECKBOX FIELDS X

ABOUT THE SCREENING SITE

*1a	Name of Country:	*1b. Name of City/Town/Village:
2	Site ID and / or investigator email address:	
3	Where is your screening site?	☐ Hospital/Clinic/Pharmacy ☐ Workplace ☐ Public area (indoors)<br>☐ Public area (outdoors) ☐ Home ☐ Other
*4	Date of measurement	……../…….. /……..

ABOUT THE PARTICIPANT

BY COMPLETING THIS FORM YOU ARE CONSENTING TO SHARE YOUR INFORMATION FOR ACADEMIC RESEARCH PURPOSES
IF YOU DO NOT KNOW THE ANSWER LEAVE BLANK

5	Ethnicity** (self-declared)	☐ Black ☐ White ☐ South Asian ☐ East/South East Asian ☐ Middle Eastern ☐ Mixed ☐ Other
6	When did you last have your blood pressure (BP) measured?	☐ Never ☐ Over 12 months ago ☐ Within the last 12 months
7	Which years have you participated in MMM before today? (Tick all that apply)	☐ Never ☐ 2017 ☐ 2018 ☐ 2019
*8	Have you ever been diagnosed with high BP by a health professional (except in pregnancy)?	☐ Yes ☐ No
9	How many drug classes are you currently taking for your BP?***	☐ 0 ☐ 1 ☐ 2 ☐ 3 ☐ 4 ☐ 5 + ☐ Don't know
10	Do you usually pay fees for your consultations and/or medications when you get your BP treated?	☐ Pay nothing ☐ Pay part ☐ Pay fully ☐ Not sure if part or fully paid
11	Do you take your BP medication regularly? If not - why? (Tick all that apply)	☐ I do ☐ Too expensive ☐ Not easily available ☐ Side effects<br>☐ Only take them when I need them ☐ Prefer alternative medicine ☐ I forget
12	Are you currently taking the following medications?	**a)** Statin ☐ Yes ☐ No ☐ Don't know **b)** Aspirin ☐ Yes ☐ No ☐ Don't know
*13	How old are you in years? (Estimate if unknown)	yrs ☐ Mark with X if estimated
*14	What is your sex?	☐ Male ☐ Female ☐ Other
15	If female, are you pregnant?	☐ Yes ☐ No
16	If female, have you had raised BP in this or a previous pregnancy?	☐ Yes ☐ No
17	If female, are you currently taking…. **a)** Hormonal contraception ☐ Yes ☐ No	**b)** Hormone replacement treatment (HRT) ☐ Yes ☐ No
18	Do you use tobacco?	☐ Yes ☐ No – but I did in the past ☐ Never
19	Do you consume alcohol?	☐ Never/rarely ☐ 1-3 times per month ☐ 1-6 times per week ☐ Daily
20	Have you been diagnosed as having diabetes by a health professional (except in pregnancy)?	☐ Yes ☐ No
21	Have you ever suffered from a….	**a)** Heart attack ☐ Yes ☐ No **b)** Stroke ☐ Yes ☐ No
22	Have you had a positive test for COVID-19 (Coronavirus)?	☐ Yes ☐ No
23	How was your BP treatment affected by COVID-19 (Coronavirus)?	☐ Not at all or N/Aǂ ☐ Usual drug(s) unavailable ☐ Old drug(s) changed<br>☐ New drug(s) added ☐ Began drug(s) for the first time ☐ Stopped my drugs
24	Have you received the COVID-19 vaccination?	☐ Yes ☐ No
25	Do you take part in at least 150mins of moderate exercise (brisk walking) or 75 mins of more vigorous exercise per week?	☐ Yes ☐ No
26	How many years of education do you have?	☐ 0 ☐ 1-6 years ☐ 7-12 years ☐ over 12 years

MEASUREMENTS

27	Weight (estimate if not measured)	Kilograms (kg) **OR** Pounds (lbs)	☐ Mark with X if estimated
28	What was your birthweight?	Kilograms (kg) **OR** Pounds (lbs)	☐ Don't know
29	What is the manufacturer of the BP machine being used?	☐ OMRON ☐ Other	

		Systolic Blood Pressure (SBP)	Diastolic Blood Pressure (DBP)	Pulse
*30	1st measurement			
	2nd measurement			
	3rd measurement			

*These questions must be answered in order to be submitted for May Measurement Month

NB: Do not record any personal data that would identify the patient e.g. name, address.

** **South Asian** – with origins from: India, Pakistan, Bangladesh, Nepal, Bhutan, Maldives and Sri Lanka. **East and South-East Asian** – With Origins from any countries east of the Indian sub-continent.
*** This means how many types of medications are being taken i.e. – ACE-inhibitors, ARBs, diuretics, beta-blockers, calcium channel blockers, alpha-blockers, others. If you are unsure, please enter the number of different tablets each day. (If you are taking 1 tablet twice a day, this counts as 1).

ǂ N/A = Not applicable

MAY MEASURE MONTH

OUR TOP TIPS
FOR HEALTHY BLOOD PRESSURE

THANK YOU FOR BEING PART OF MAY MEASUREMENT MONTH.

For more information visit www.maymeasure.org

If you have concerns, please seek advice from a trained medical professional.

MAINTAIN A HEALTHY WEIGHT

Just doing this can help bring down high blood pressure.

EXERCISE REGULARLY

Aim for an average of 30 minutes a day. For the exercise to be worthwhile, you need to feel warmer, breathe harder, and your heart needs to beat faster than it normally does.

EAT PLENTY OF FRUIT AND/OR VEGETABLES EVERY DAY

Eat vegetables raw or lightly steamed, rather than boiled, to get maximum nutrition. Avoid frying where possible.

ADD BEETROOT TO YOUR DIET

Regular consumption of beetroot juice has been found to help reduce your blood pressure.

CUT DOWN ON SALT

Reduce your intake of salt. Don't forget a lot of salt is hidden in processed foods and is very high in most breads, cereals, soups and sauces. If possible, always read the label. Eating a low-fat diet that includes lots of fibre, such as bread, pasta, rice and wholegrains has also been found to help reduce blood pressure.

CUT DOWN ON FAT AND SUGAR

Always check the label on foods where possible and be especially wary of hydrogenated or 'trans' fats, as well as sugars 'hidden' as other names such as sucrose, dextrose, fructose, and glucose. These can be commonly found in items such as fruit juices and fizzy drinks.

STOP SMOKING TOBACCO

Your arteries clog up even faster if you smoke and this causes many other health problems. Your blood pressure actually rises while you smoke.

REDUCE YOUR CAFFEINE INTAKE

Remember caffeine is found in some fizzy drinks as well as in coffee and tea.

DON'T DRINK TOO MUCH ALCOHOL

Stick to local daily recommendations – usually less than 2 drinks for men and 1 for women (1 drink = small beer or wine).

REGULAR CHECK UPS

Have your blood pressure measured by a trained medical professional regularly.

RELAX

Stress contributes to raising blood pressure. So, avoid stress where possible and allow time for relaxation.

#TheBigSqueeze

www.facebook.com/MayMeasure
@MayMeasureOrg
maymeasureorg

Data cleaning rules

Rules for cleaning continuous variables and dates.
Updated January 2022

Weight (weight and scale variables)
Upper limit: 160.0kg (25.2 stone, 353lbs)
Lower limit: 30.0kg (4.72 stone, 66lbs)

Extract measurement scale from measurement string if missing
Assume recorded in kg if unit of measurement missing

Convert all values to kg, then correct values:
Replace weight/1,000 if weight > 30,000
Replace weight/100 if weight > 3,000
Replace weight/10 if weight > 300
Exclude values > 160 or < 30

Birth weight
Upper limit: 5.5kg (based on Blencowe *et al* (2019))[1]
Lower limit: 1.0kg

Extract measurement scale from measurement string if missing
Assume birth weight recorded in kg if unit of measurement missing
Replace birth weight/1,000 if birth weight > 1,000

Survey date
Assume format DD/MM/YYYY as specified on template
If month ≥ 13 then switch D & M
Exclude dates where day > 31, or >30 in months of April, June, September or November, or >28 in February

Age
Upper limit: 99 years (set ages > 99 to missing)
Lower limit: 18 years (drop participants < 18)

Set entries with age > 99 to missing
Exclude participants from study if age recorded as < 18

Systolic blood pressure
Upper limit: 260 mmHg
Lower limit: 80 mmHg

Multiply by 10 entries ≥ 8 and ≤ 26
Remove first digit in entries of format 11XX (assume 1 double-typed)
Divide by 10 entries ≥ 1,200 & ≤ 2,600
Exclude entries outside of above range

Diastolic blood pressure
Upper limit: 160 mmHg
Lower limit: 40 mmHg

Multiply by 10 entries ≥ 5 and ≤ 15
Remove first digit in entries of format 11XX (assume 1 double-typed)
Divide by 10 entries ≥ 500 & ≤ 1,500
Exclude entries outside of above range

Drop SBP and DBP if DBP ≥ SBP

Heart rate
Upper limit: 120 bpm
Lower limit: 30 bpm

Multiply by 10 entries ≥ 3 and ≤ 12

Remove first digit in entries of format 11XX
Divide by 10 entries ≥ 300 & ≤ 1,200
Exclude entries outside of above range

Sex and pregnancy/hypertension in pregnancy/hormone replacement therapy/hormonal contraception
Set pregnancy to missing where gender = 'male' or 'other'
If gender missing and pregnancy/hypertension in previous pregnancy/hormone replacement therapy/hormonal contraception = 'yes', assume gender = 'female'

Known hypertension and medication
Assume known hypertension = 'yes' if medication = 'yes'

Antihypertensive medication status and number of medication classes
If medication status = 'none' then set medication classes to '0'
If medication = 'yes' and medication classes = '0', replace medication classes as missing
If medication classes = 0 and medication status missing, replace medication status to 'none'; if medication classes ≥ 1 and medication status missing, set medication status to 'yes'

Statistical analysis

Multiple imputation

Of 642,057 participants in MMM21, 85.5% had all three blood pressure (BP) readings, 8.8% had two readings and 5.7% had a single reading (Table S1).

Table S1: Number of BP readings per participant

Number of BP readings per participant	Total	Percentage
1	36,715	5.7%
2	56,359	8.8%
3	548,983	85.5%

Given the tendency for BP readings to fall on average across subsequent clinic BP readings (Table S4), we imputed missing readings to allow for more robust comparison for those with one or more readings missing. Multiple imputation using chained equations was used, under the assumption that the pattern of missing BP readings was missing at random dependent on the observed data.

Two separate imputation models were run. The first, 'full' model was conducted for those with no missing data in age, sex (excluding the 'other' category due to small numbers), ethnicity and antihypertensive medication use (yes/no). Included in the model were: age, sex, an interaction between age and sex, ethnicity, antihypertensive medication use, known hypertension, diabetes, myocardial infarction, stroke, alcohol intake, smoking status, weight, screening site type along with each of the three systolic BP, diastolic BP and heart rate (HR) measurements, and the mean of the second and third BP and HR readings. Age was included as a restricted cubic spline with five knots to allow flexibility to modelling its relationship and weight was included along with a quadratic term.

A second imputation was run for those participants that could not be imputed using the full model (due to missing one or more of age, sex, ethnicity or antihypertensive medication use). The second, 'partial' model included each systolic BP and diastolic BP reading, along with the mean of the second and third BP readings.

For each imputation model, fifteen imputations were created, corresponding to the approximate percentage of missing data in the mean of the second and third BP readings. The Monte Carlo errors of estimates were <10% of their standard errors indicating sufficient precision.[2] A burn-in of ten iterations was deemed sufficient based on convergence of trace plots. Results from each imputation set were combined using Rubin's combination rules.

For the primary analysis, a combined model was created, which used imputations from the full model where available, but for those individuals with missing BP readings that could not be imputed from the full model, used the partial imputation model. Sensitivity analyses were conducted comparing the complete cases analysis to each of the imputation models:

1. The complete case sample only (those with a mean of BP readings 2 and 3).
2. The partial multiple imputation model including BP readings for all participants.
3. The full multiple imputation model as described above for individuals with complete data on age, gender, ethnicity and anti-hypertensive medication use.
4. The combined model, including imputations for individuals from the partial model, where readings could not be imputed from the full model.

Results were similar across the imputation models, and the combined model was used as the primary analysis (Table S5).

Measures of association

Measures of association between systolic or diastolic BP with different risk factors were examined using linear mixed models, for those with complete data on age, sex, ethnicity and antihypertensive medication use (i.e., for those included in the full imputation model), including the country in which the participant was screened was included as a random intercept, to account for country-level clustering. Models were adjusted for age, sex and antihypertensive medication use, including also an interaction between age and sex. Age was modelled as a restricted cubic spline with five knots, as in the full imputation model.

Models including known hypertension status and antihypertensive medication use as the exposure used only the subset of individuals with hypertension. Models using known hypertension or antihypertensive medication use as exposures were adjusted for age and sex alone. Models using pregnancy, hypertension in a previous pregnancy, hormonal contraception use and hormone replacement therapy use were adjusted for age and antihypertensive medication use alone.

Analyses was conducted using Stata version 16.1. The *mi impute chained* command was used for multiple imputation, and the *mixed* command for linear mixed models.

References:

1. Blencowe, H. et al. National, regional, and worldwide estimates of low birthweight in 2015, with trends from 2000: a systematic analysis. Lancet Glob. Heal. 7, e849–e860 (2019).
2. White IR, Royston P, Wood AM, Multiple imputation using chained equations: Issues and guidance for practice. *Statistics in Medicine*. 2010: 30:4.

Table S2: Participant mean age, sex distribution and percentage on antihypertensive medication by region and country

Region and country	Total participants	Percentage	Mean age (SD) in years	Females*	Males*	On anti-hypertensive medication
**East Asia**	**222,614**	**34.7%**	47.1 (15.6)	114832 (51.6%)	107722 (48.4%)	18097 (8.1%)
China	218,844	34.1%				
Taiwan	3,738	0.6%				
Mongolia	32	<0.1%				
**Americas**	**156,513**	**24.4%**	50.4 (16.8)	93310 (59.9%)	62528 (40.1%)	35953 (23.0%)
Mexico	77,548	12.1%				
Venezuela	46,732	7.3%				
Argentina	26,070	4.1%				
Paraguay	3,633	0.6%				
Ecuador	1,326	0.2%				
Jamaica	526	0.1%				
Brazil	340	0.1%				
Guatemala	338	0.1%				
**South-east Asia and Australasia**	**89,631**	**14.0%**	43.2 (16.4)	89631 (14.0%)	44288 (49.9%)	16613 (18.5%)
Philippines	61,129	9.5%				
Indonesia	13,452	2.1%				
Thailand	10,940	1.7%				
Vietnam	2,572	0.4%				
Australia	1,348	0.2%				
Malaysia	190	<0.1%				
**Sub-Saharan Africa**	**73,880**	**11.5%**	40.5 (16.3)	35422 (48.0%)	38300 (51.9%)	17777 (24.1%)
Democratic Republic of the Congo	20,913	3.3%				
Niger	11,960	1.9%				
Kenya	9,738	1.5%				
Nigeria	9,361	1.5%				
Malawi	7,074	1.1%				
Ghana	4,837	0.8%				
South Africa	2,294	0.4%				
Republic of the Congo	2,135	0.3%				
Zimbabwe	2,094	0.3%				
Uganda	1,671	0.3%				
Cabo Verde	1,214	0.2%				
Zambia	418	0.1%				

Mali	171	<0.1%				
**South Asia**	**59,946**	**9.3%**	42.6 (14.8)	22637 (37.9%)	37152 (62.1%)	11879 (19.8%)
Bangladesh	28,355	4.4%				
India	15,063	2.3%				
Pakistan	11,367	1.8%				
Nepal	5,161	0.8%				
**Europe**	**38,327**	**6.0%**	50.8 (17.4)	22719 (59.3%)	15550 (40.6%)	12886 (33.6%)
Albania	11,788	1.8%				
Armenia	5,777	0.9%				
Georgia	4,935	0.8%				
Bulgaria	3,908	0.6%				
Russia	2,644	0.4%				
Spain	2,012	0.3%				
Kazakhstan	1,763	0.3%				
Poland	1,704	0.3%				
Hungary	1,414	0.2%				
United Kingdom	764	0.1%				
Republic of Ireland	568	0.1%				
Kyrgyzstan	359	0.1%				
North Macedonia	245	<0.1%				
Slovakia	197	<0.1%				
Switzerland	120	<0.1%				
Lithuania	98	<0.1%				
Denmark	31	<0.1%				
**Northern Africa and Middle East**	**1,146**	**0.2%**	47.1 (16.7)	706 (61.6%)	440 (38.4%)	375 (32.7%)
Sudan	487	0.1%				
Iran	400	0.1%				
Algeria	259	<0.1%				
**Worldwide**	**642,057**		**46.4 (16.5)**	**333965 (52.2%)**	**305980 (47.8%)**	**113580 (17.7%)**

Table S3: Participant characteristics screened in MMM21

Participant characteristic		Total participants	Percentage including unknown	Percentage not including unknown
**Sex**	Female	333,965	52.0%	52.2%
	Male	305,980	47.7%	47.8%
	Other	290	<0.1%	<0.1%
	Unknown	1,822	0.3%	
**Age (years)**	Mean (SD)	46.4 (16.5)		
	18-29	121,671	19.0%	19.1%
	30-39	115,660	18.0%	18.1%
	40-49	121,071	18.9%	19.0%
	50-59	129,556	20.2%	20.3%
	60-69	91,534	14.3%	14.3%
	70 or more	58,648	9.1%	9.2%
	Unknown	3,917	0.6%	
**Ethnicity**	East/south-east Asian	266,325	41.5%	52.6%
	Black	75,572	11.8%	14.9%
	South Asian	68,945	10.7%	13.6%
	White	51,094	8.0%	10.1%
	Other	40,726	6.3%	8.0%
	Mixed	3,347	0.5%	0.7%
	Middle Eastern	308	0.0%	0.1%
	Unknown	136,186	21.2%	
**Country income group**	Low income	42,276	6.6%	6.6%
	Lower middle income	167,946	26.2%	26.2%
	Upper middle income	419,841	65.4%	65.4%
	High income	11,994	1.9%	1.9%
**On anti-hypertensive medication**	No	283,061	44.1%	71.4%
	Yes	113,580	17.7%	28.6%
	Unknown	245,416	38.2%	
**Number of anti-hypertensive medication classes**	0	283,061	44.1%	73.4%
	1	59,652	9.3%	15.5%
	2	28,218	4.4%	7.3%
	3	10,569	1.6%	2.7%
	4	3,425	0.5%	0.9%
	5 or more	809	0.1%	0.2%
	Unknown	256,323	39.9%	
**Regularity of anti-hypertensive medication use**	I do	64,720	10.1%	53.5%
	Too expensive	5,892	0.9%	4.9%
	Not easily available	3,729	0.6%	3.1%
	Side effects	448	0.1%	0.4%
	Only take them when I need them	43,097	6.7%	35.6%
	Prefer alternative medicine	919	0.1%	0.8%
	I forget	2,151	0.3%	1.8%
	Unknown	521,101	81.2%	
**Location of screening site**	Hospital/clinic/pharmacy	331,054	51.6%	65.7%
	Public area (outdoors)	77,439	12.1%	15.4%
	Public area (indoors)	25,434	4.0%	5.0%
	Workplace	30,506	4.8%	6.1%
	Other	24,975	3.9%	5.0%

	Vaccination site	14,779	2.3%	2.9%
	Unknown	137,870	21.5%	
**Participated in MMM 2017/2018/2019**	No	208,226	32.4%	46.7%
	Yes	237,519	37.0%	53.3%
	Unknown	196,312	30.6%	
**Last BP measurement**	Never	53,180	8.3%	15.5%
	More than 12 months	79,416	12.4%	23.1%
	Within 12 months	211,405	32.9%	61.5%
	Unknown	298,056	46.4%	
**Diabetes**	No	444,377	69.2%	94.8%
	Yes	24,382	3.8%	5.2%
	Unknown	173,298	27.0%	
**Previous MI**	No	468,651	73.0%	97.8%
	Yes	10,535	1.6%	2.2%
	Unknown	162,871	25.4%	
**Previous stroke**	No	471,594	73.5%	98.7%
	Yes	6,230	1.0%	1.3%
	Unknown	164,233	25.6%	
**On aspirin**	No	429,459	66.9%	93.4%
	Yes	30,313	4.7%	6.6%
	Unknown	182,285	28.4%	
**On a statin**	No	427,642	66.6%	92.9%
	Yes	32,460	5.1%	7.1%
	Unknown	181,955	28.3%	
**Pregnant***	No	328,949	98.5%	98.5%
	Yes	5,016	1.5%	1.5%
**Hypertensive in previous pregnancy***	No	327,083	97.9%	97.9%
	Yes	6,882	2.1%	2.1%
**Hormonal contraception use***	No	325,845	97.6%	98.7%
	Yes	8,120	2.4%	1.3%
**Hormone replacement treatment (HRT) use***	No	331,875	99.4%	99.4%
	Yes	2,090	0.6%	0.6%
**Current smoker**	No	440,007	68.5%	90.1%
	Yes	48,385	7.5%	9.9%
	Unknown	153,665	23.9%	
**Alcohol intake**	Never/rarely	420,152	65.4%	87.4%
	1-3 times per month	47,492	7.4%	9.9%
	1-6 times per week	13,264	2.1%	2.8%
	Daily	5,473	0.9%	1.1%
	Unknown	155,676	24.2%	
**Weight (kg)**	Mean (SD)	67.1 (13.3)		
	<50 kg	28,271	4.4%	5.5%
	50-59 kg	118,626	18.5%	23.0%
	60-69 kg	171,510	26.7%	33.2%
	70-79 kg	117,140	18.2%	22.7%
	80-89 kg	51,696	8.1%	10.0%
	90-99 kg	17,849	2.8%	3.5%
	100 kg or more	11,114	1.7%	2.2%
	Unknown	125,851	19.6%	
**Years of formal education**	0 year	19,625	3.1%	4.6%

	1-6 years	97,188	15.1%	22.9%
	7-12 years	167,787	26.1%	39.5%
	Over 12 years	140,356	21.9%	33.0%
	Unknown	217,101	33.8%	
**Meeting WHO physical activity recommendations**	No	335,871	52.3%	72.3%
	Yes	128,505	20.0%	27.7%
	Unknown	177,681	27.7%	
**Payment for consultations/medications**	Pay nothing	83,273	13.0%	19.6%
	Pay part	38,340	6.0%	9.0%
	Pay fully	44,646	7.0%	10.5%
	Unknown	475,798	74.1%	
**Previous positive test for COVID-19**	No	450,903	70.2%	94.8%
	Yes	24,614	3.8%	5.2%
	Unknown	166,540	25.9%	
**How BP treatment affected by COVID-19**	Not at all or N/A	381,996	59.5%	97.9%
	Usual drug(s) unavailable	2,934	0.5%	0.8%
	Old drug(s) changed	807	0.1%	0.2%
	New drug(s) added	2,066	0.3%	0.5%
	Began drug(s) for the first time	438	0.1%	0.1%
	Stopped my drugs	482	0.1%	0.1%
	Unable to access healthcare provider	1,653	0.3%	0.4%
	Unknown	251,681	39.2%	
**Previous COVID-19 vaccination**	No	255,919	39.9%	54.8%
	Yes	210,839	32.8%	45.2%
	Unknown	175,299	27.3%	
**Manufacturer of the BP device used**	Omron	447,749	69.7%	90.1%
	Other	49,252	7.7%	9.9%
	Unknown	145,056	22.6%	
**Of Omron devices, model of device**	Omron HEM-7081-IT	217,475	33.9%	33.9%
	Omron JPN1	15,045	2.3%	2.3%
	Omron M2 Basic	6,847	1.1%	1.1%
	Omron M3 Intellisense	962	0.1%	0.1%
	Omron device NOS	207,420	32.3%	32.3%
**Day of week of screening**	Monday	94,605	14.7%	15.6%
	Tuesday	89,547	13.9%	14.7%
	Wednesday	106,572	16.6%	17.5%
	Thursday	100,041	15.6%	16.4%
	Friday	99,399	15.5%	16.3%
	Saturday	68,122	10.6%	11.2%
	Sunday	49,987	7.8%	8.2%
	Unknown	33,784	5.3%	
**Total participants**		**642,057**		

* Percentage given of females only

Table S4: Mean BP and corresponding number and proportion of participants with hypertension based on the mean of each reading (of 1,133,008 participants with all 3 readings

BP reading	Mean systolic BP (mmHg)	Mean diastolic BP (mmHg)	Number with hypertension	Percentage with hypertension
1	126.3	80.4	211,257	32.9%
2	124.4	79.2	195,328	30.4%
3	123.5	78.6	187,138	29.1%
Mean of 1 & 2	125.6	80.0	197,540	30.8%
**Mean of 2 & 3**	**124.2**	**79.1**	**186,820**	**29.1%**
Mean of 1 & 2 & 3	124.7	79.4	187,668	29.2%

Table S5: Mean systolic and diastolic BP and percentages with raised BP comparing the complete case to the partial, full and combined imputation models

Model	Total in analysis	Mean systolic BP (mmHg)	Mean diastolic BP (mmHg)	Percentage with raised BP		
				Overall	Not on medication	On medication
Complete case	552,080*	124.19	79.08	25.39%	20.64%	46.26%
Partial model	642,057	124.54	79.14	25.71%	20.55%	46.33%
Full model	574,668*	124.27	79.15	25.62%	20.43%	46.34%
Combined model	642,057	124.50	79.12	25.64%	20.51%	46.09%

*Combined MI model is the primary imputation model used in analysis

Table S6: Worldwide and regional confidence intervals for estimates of percentage with hypertension, awareness, on treatment and controlled (of all 642,057 participants)

Region	Percentage with hypertension	Percentage of hypertensives aware	Percentage of hypertensives on medication	Percentage of those on medication with controlled BP	Percentage of all hypertensives controlled
East Asia	(26.4% , 26.7%)	(32.1% , 32.7%)	(30.4% , 30.8%)	(60.8% , 62.2%)	(18.5% , 19.1%)
Americas	(38.4% , 38.9%)	(72.1% , 73.7%)	(59.1% , 59.8%)	(54.4% , 55.5%)	(32.3% , 33.1%)
South-east Asia and Australasia	(40.8% , 41.5%)	(49.0% , 50.2%)	(44.7% , 45.4%)	(46.0% , 47.5%)	(20.7% , 21.5%)
Sub-Saharan Africa	(37.5% , 38.2%)	(65.8% , 67.6%)	(63.0% , 64.2%)	(51.2% , 52.7%)	(32.4% , 33.6%)
South Asia	(35.2% , 36.0%)	(63.2% , 66.0%)	(55.0% , 56.2%)	(56.7% , 58.6%)	(31.4% , 32.7%)
Europe	(50.4% , 51.4%)	(70.1% , 72.3%)	(65.4% , 66.7%)	(48.2% , 50.0%)	(31.8% , 33.1%)
Northern Africa and Middle East	(47.1% , 52.9%)	(64.5% , 76.0%)	(61.8% , 69.4%)	(34.7% , 44.7%)	(22.4% , 29.6%)
**Worldwide**	(35.1% , 35.3%)	(56.6% , 57.1%)	(50.1% , 50.5%)	(53.6% , 54.2%)	(26.9% , 27.3%)

Table S7: Number and percentage of participants hypertension according to age group

Age category (years)	Number with hypertension	Percentage with hypertension	Denominator
18-29	16,777	13.8%	121,671
30-39	26,921	23.3%	115,660
40-49	40,452	33.4%	121,071
50-59	55,169	42.6%	129,556
60-69	48,588	53.1%	91,534
70 or more	36,322	61.9%	58,648
Missing	1,653	42.2%	3,917

Table S8: Number and percentage of participants with hypertension according to sex and age group

Age category (years)	Female					Male				
	Number with hypertension	Percentage with hypertension	95% confidence interval		Denominator	Number with hypertension	Percentage with hypertension	95% confidence interval		Denominator
			Lower	Upper				Lower	Upper	
18-29	7,618	12.2%	12.0%	12.5%	62,284	9,082	15.4%	15.1%	15.7%	58,963
30-39	13,100	21.7%	21.3%	22.0%	60,427	13,723	25.0%	24.6%	25.4%	54,929
40-49	19,490	30.5%	30.2%	30.9%	63,854	20,834	36.7%	36.3%	37.1%	56,840
50-59	28,371	41.9%	41.5%	42.2%	67,785	26,642	43.4%	43.0%	43.8%	61,407
60-69	25,084	52.8%	52.4%	53.3%	47,506	23,432	53.5%	53.0%	53.9%	43,830
70 or more	19,328	63.8%	63.2%	64.3%	30,303	16,942	60.0%	59.4%	60.6%	28,249
Missing	811	44.9%	42.5%	47.2%	1,806	672	38.2%	35.9%	40.5%	1,762

Table S9: Numbers with hypertension and parameters of awareness, treatment and control stratified by sex

Sex	Number with hypertension	Percentage with hypertension	Percentage of hypertensives aware	Percentage of hypertensives on medication	Percentage of those on medication with controlled BP	Percentage of all hypertensives controlled
Female	113,802	34.1%	62.8%	55.8%	56.2%	31.4%
Male	111,327	36.4%	50.7%	44.8%	51.0%	22.9%

Table S10: Worldwide and regional numbers with hypertension and parameters of awareness, treatment and control after age and sex standardisation (of 636,658 participants with age and sex recorded)

Region	Number with hypertension	Percentage with hypertension	Percentage of hypertensives aware	Percentage of hypertensives on medication	Percentage of those on medication with controlled BP	Percentage of all hypertensives controlled
East Asia	52,195	23.5%	32.4%	24.2%	53.9%	13.1%
Americas	46,727	30.1%	77.5%	37.1%	59.5%	22.0%
South-east Asia and Australasia	34,336	39.0%	50.6%	36.1%	57.7%	20.8%
Sub-Saharan Africa	26,834	36.5%	62.9%	51.5%	55.5%	28.6%
South Asia	19,026	32.8%	60.9%	45.6%	62.8%	28.6%
Europe	15,670	41.1%	70.6%	42.0%	61.1%	25.6%
Northern Africa and Middle East	520	45.4%	69.1%	58.7%	35.6%	20.9%
**Worldwide**	**197,852**	**31.1%**	**56.2%**	**38.0%**	**59.9%**	**22.8%**

Measures of association

Table S11: Results from linear mixed models comparing mean systolic and diastolic BP in those with each risk factor compared to those without, adjusted for age, sex, of participants with hypertension

Blood pressure	Risk factor	Mean BP difference compared to absence of risk factor (mmHg)	Monte Carlo error of estimate	Standard error	t value	p value	95% confidence interval		Number in analysis†
							Lower bound	Upper bound	
Systolic	Known hypertension§	-8.923	0.004	0.093	-95.660	<0.001	-9.105	-8.740	217,750
Systolic	At least one antihypertensive medication*	-12.517	0.006	0.120	-104.590	<0.001	-12.751	-12.282	169,406
Systolic	Number of antihypertensive classes: 1*	-13.323	0.005	0.132	-101.030	<0.001	-13.582	-13.065	158,500
Systolic	Number of antihypertensive classes: 2*	-13.026	0.004	0.153	-85.130	<0.001	-13.326	-12.726	158,500
Systolic	Number of antihypertensive classes: 3*	-6.920	0.006	0.215	-32.230	<0.001	-7.341	-6.500	158,500
Systolic	Number of antihypertensive classes: 4*	-6.100	0.011	0.333	-18.330	<0.001	-6.752	-5.447	158,500
Systolic	Number of antihypertensive classes: 5 or more*	-10.410	0.026	0.645	-16.140	<0.001	-11.674	-9.146	158,500
Diastolic	Known hypertension§	-6.389	0.003	0.059	-107.970	<0.001	-6.505	-6.273	217,750
Diastolic	At least one antihypertensive medication*	-9.627	0.003	0.074	-129.390	<0.001	-9.773	-9.481	169,406
Diastolic	Number of antihypertensive classes: 1*	-10.159	0.001	0.082	-123.250	<0.001	-10.321	-9.998	158,500
Diastolic	Number of antihypertensive classes: 2*	-9.729	0.004	0.097	-100.260	<0.001	-9.919	-9.539	158,500
Diastolic	Number of antihypertensive classes: 3*	-7.234	0.005	0.136	-53.300	<0.001	-7.500	-6.968	158,500
Diastolic	Number of antihypertensive classes: 4*	-7.179	0.009	0.211	-34.090	<0.001	-7.592	-6.766	158,500
Diastolic	Number of antihypertensive classes: 5 or more*	-9.697	0.014	0.405	-23.940	<0.001	-10.491	-8.903	158,500

§ Adjusted in addition for antihypertensive medication use
* Compared to those on no antihypertensive medication
† Numbers in analysis vary as dependent on the number of individuals with complete data on each risk factor

Table S12: Results from linear mixed models comparing mean systolic and diastolic BP for participants with a previous positive COVID-19 test or vaccination, adjusted for age, sex and antihypertensive medication

Blood pressure	Risk factor	Mean BP difference compared to absence of factor (mmHg)	Monte Carlo error of estimate	Standard error	t value	p value	95% confidence interval		Number in analysis†
							Lower bound	Upper bound	
Systolic	Positive test for COVID-19	-0.273	0.006	0.161	-1.690	0.091	-0.589	0.043	357,976
Systolic	Received at least one COVID-19 vaccination	-0.684	0.001	0.066	-10.360	<0.001	-0.813	-0.554	355,307
Diastolic	Positive test for COVID-19	0.318	0.005	0.106	3.000	0.003	0.110	0.527	357,976
Diastolic	Received at least one COVID-19 vaccination	-0.935	0.001	0.043	-21.530	<0.001	-1.020	-0.850	355,307

† Numbers in analysis vary as dependent on the number of individuals with complete data on each risk factor

Table S13: Results from linear mixed models comparing mean systolic and diastolic BP in those with each risk factor compared to those without, adjusted for age, sex and antihypertensive medication.

Blood pressure	Risk factor	Mean BP difference compared to absence of risk factor (mmHg)	Monte Carlo error of estimate	Standard error	t value	p value	95% confidence interval		Number in analysis†
							Lower bound	Upper bound	
Systolic	Current smoker (vs non-smoker)	0.272	0.003	0.104	2.630	0.009	0.069	0.475	368,245
Systolic	Alcohol: 1-3 times per month§	-0.380	0.004	0.105	-3.630	<0.001	-0.585	-0.174	368,208
Systolic	Alcohol: 1-6 times per week§	1.372	0.008	0.199	6.880	<0.001	0.981	1.763	368,208
Systolic	Alcohol: daily§	2.002	0.010	0.301	6.650	<0.001	1.412	2.592	368,208
Systolic	Education: 1-6 years*	1.472	0.004	0.153	9.650	<0.001	1.173	1.772	337,023
Systolic	Education: 7-12 years*	-0.168	0.003	0.151	-1.120	0.264	-0.463	0.127	337,023
Systolic	Education: over 12 years*	-2.355	0.003	0.158	-14.930	<0.001	-2.664	-2.045	337,023
Systolic	Physical activity: 150 mins of moderate or 75 mins of more vigorous per week	-1.418	0.002	0.078	-18.250	<0.001	-1.570	-1.265	353,826
Diastolic	Current smoker (vs non-smoker)	0.641	0.002	0.068	9.410	<0.001	0.507	0.774	368,245
Diastolic	Alcohol: 1-3 times per month§	0.572	0.003	0.069	8.240	<0.001	0.436	0.708	368,208
Diastolic	Alcohol: 1-6 times per week§	3.078	0.004	0.130	23.680	<0.001	2.824	3.333	368,208
Diastolic	Alcohol: daily§	2.581	0.005	0.197	13.130	<0.001	2.196	2.967	368,208
Diastolic	Education: 1-6 years*	-0.571	0.003	0.101	-5.680	<0.001	-0.768	-0.374	337,023
Diastolic	Education: 7-12 years*	-0.744	0.003	0.099	-7.500	<0.001	-0.939	-0.550	337,023
Diastolic	Education: over 12 years*	-1.886	0.002	0.104	-18.160	<0.001	-2.089	-1.682	337,023
Diastolic	Physical activity: 150 mins of moderate or 75 mins of more vigorous per week	-1.138	0.001	0.051	-22.370	<0.001	-1.238	-1.038	353,826

§ Compared with ‘never/rare’ alcohol use
* Compared with ‘0 years’ of education
† Numbers in analysis vary as dependent on the number of individuals with complete data on each risk factor

Table S14: Results from linear mixed models comparing mean systolic and diastolic BP in those with diabetes, previous myocardial infarction or stroke compared to those without, adjusted for age, sex and antihypertensive medication.

Blood pressure	Co-morbidity	Mean BP difference compared to absence of condition (mmHg)	Monte Carlo error of estimate	Standard error	t value	p value	95% confidence interval		Number in analysis†
							Lower bound	Upper bound	
Systolic	Diabetes	-0.309	0.011	0.148	-2.100	0.036	-0.599	-0.020	367,810
Systolic	Previous Myocardial Infarction	-0.217	0.011	0.201	-1.080	0.280	-0.610	0.177	368,001
Systolic	Previous Stroke	0.175	0.019	0.261	0.670	0.503	-0.337	0.686	368,129
Diastolic	Diabetes	-0.897	0.004	0.095	-9.490	<0.001	-1.083	-0.712	367,810
Diastolic	Previous Myocardial Infarction	-1.425	0.009	0.134	-10.670	<0.001	-1.687	-1.163	368,001
Diastolic	Previous Stroke	0.058	0.011	0.169	0.350	0.730	-0.273	0.390	368,129

† Numbers in analysis vary as dependent on the number of individuals with complete data on each risk factor

Table S15: Results from linear mixed models comparing mean systolic and diastolic BP in females with pregnancy, previous hypertension in pregnancy, hormone replacement therapy or hormonal contraception use, adjusted for age and antihypertensive medication

Blood pressure	Risk factor	Mean BP difference compared to absence of risk factor (mmHg)	Monte Carlo error of estimate	Standard error	t value	p value	95% confidence interval		Number in analysis†
							Lower bound	Upper bound	
Systolic	Current pregnancy	-4.654	0.007	0.339	-13.720	<0.001	-5.319	-3.990	213,121
Systolic	Hypertension in previous pregnancy	2.535	0.008	0.275	9.230	<0.001	1.997	3.073	213,121
Systolic	Hormone Replacement Therapy use	-1.163	0.011	0.517	-2.250	0.025	-2.176	-0.149	213,121
Systolic	Hormonal contraception use	0.323	0.016	0.307	1.050	0.294	-0.280	0.925	213,121
Diastolic	Current pregnancy	-4.202	0.006	0.217	-19.330	<0.001	-4.628	-3.776	213,121
Diastolic	Hypertension in previous pregnancy	2.570	0.006	0.176	14.620	<0.001	2.225	2.914	213,121
Diastolic	Hormone Replacement Therapy use	-0.100	0.012	0.333	-0.300	0.765	-0.752	0.553	213,121
Diastolic	Hormonal contraception use	0.816	0.011	0.197	4.140	<0.001	0.430	1.203	213,121

† Numbers in analysis vary as dependent on the number of individuals with complete data on each risk factor.

S16: Results from linear mixed models comparing mean systolic and diastolic BP in each heart rate category compared to heart rate in the range 40-59 bpm, adjusted for age, sex and antihypertensive medication (n=451,892)

Blood pressure	Heart rate (bpm)	Mean BP difference compared to reference heart rate (mmHg)	Monte Carlo error of estimate	Standard error	t value	p value	95% confidence interval	
							Lower bound	Upper bound
Systolic	40-59 (reference)	-	-	-	-	-	-	-
Systolic	60-69	60-69	-1.623	0.001	0.156	-10.39	<0.001	-1.930
Systolic	70-79	70-79	-1.350	0.001	0.151	-8.96	<0.001	-1.645
Systolic	80-89	80-89	-0.335	0.001	0.152	-2.2	0.028	-0.633
Systolic	90-99	90-99	1.518	0.001	0.163	9.33	<0.001	1.199
Systolic	100 or more	100 or more	4.555	0.002	0.191	23.85	<0.001	4.181
Diastolic	40-59 (reference)	-	-	-	-	-	-	-
Diastolic	60-69	60-69	1.579	0.001	0.102	15.530	<0.001	1.380
Diastolic	70-79	70-79	3.069	0.000	0.098	31.33	<0.001	2.877
Diastolic	80-89	80-89	4.781	0.000	0.099	48.26	<0.001	4.586
Diastolic	90-99	90-99	6.300	0.000	0.106	59.54	<0.001	6.093
Diastolic	100 or more	100 or more	8.196	0.001	0.124	65.99	<0.001	7.952

Figure S1: Mean systolic and blood pressure by age and sex from linear mixed models, in participants not taking antihypertensive medication

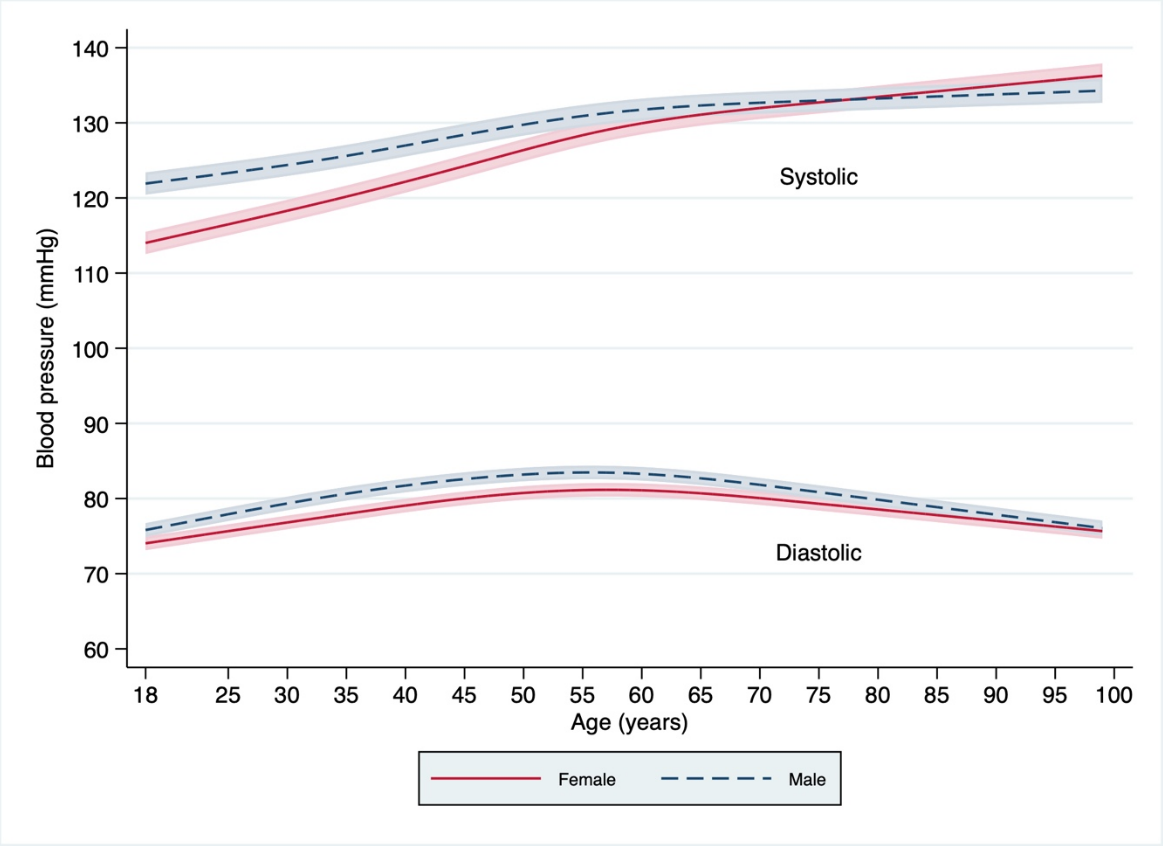

Note: shaded areas represent 95% confidence interval of estimate

Figure S2: Difference in mean systolic and diastolic BP by heart rate category compared to heart rate <60 bpm, from linear mixed models adjusted for age and sex in participants not using antihypertensive medication

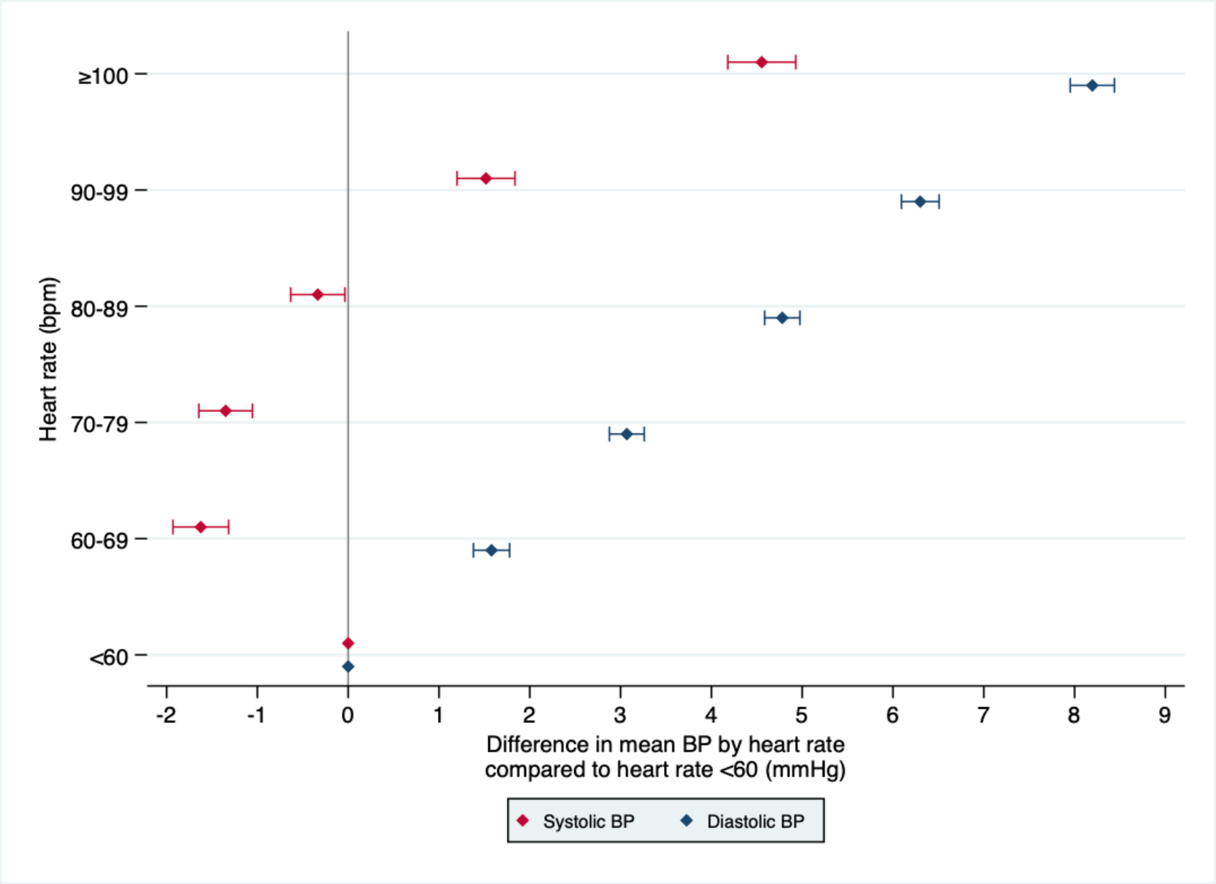

Note: diamonds represent coefficients and bars represent 95% confidence intervals of the estimate
